# Statistical machine learning for comparative protein dynamics with the DROIDS/maxDemon software pipeline

**DOI:** 10.1016/j.xpro.2022.101194

**Published:** 2022-02-24

**Authors:** Gregory A. Babbitt, Ernest P. Fokoue, Harsh R. Srivastava, Breanna Callahan, Madhusudan Rajendran

**Affiliations:** 1 Thomas H. Gosnell School of Life Sciences, Rochester Institute of Technology, Rochester, NY 14623, USA; 2 School of Mathematical Sciences, Rochester Institute of Technology, Rochester, NY 14623, USA

**Keywords:** Bioinformatics, Biophysics, Protein Biochemistry, Structural Biology, Systems biology, Computer sciences, Evolutionary biology

## Abstract

Comparative analysis of protein structure or sequence alignments often ignores the protein dynamics and function. We offer a graphical user interface to a computing pipeline, complete with molecular visualization, enabling the biophysical simulation and statistical comparison of two-state functional protein dynamics (i.e., single unbound state vs. complex with a ligand, DNA, or protein). We utilize multi-agent machine learning classifiers to identify functionally conserved dynamic motions and compare them in genetic or drug-class variants.

For complete details on the use and execution of this profile, please refer to Babbitt et al. (2020b, 2020a, 2018) and Rynkiewicz et al. (2021).

## Highlights

• A pipeline for the site-wise statistical comparison of molecular dynamics
• Analyzes mutational or functional changes in protein dynamics
• Utilizes machine learning to identify regions of conserved dynamics
• Utilizes information theoretics to identify site of coordinated dynamics

## Graphical abstract

**Figure:**
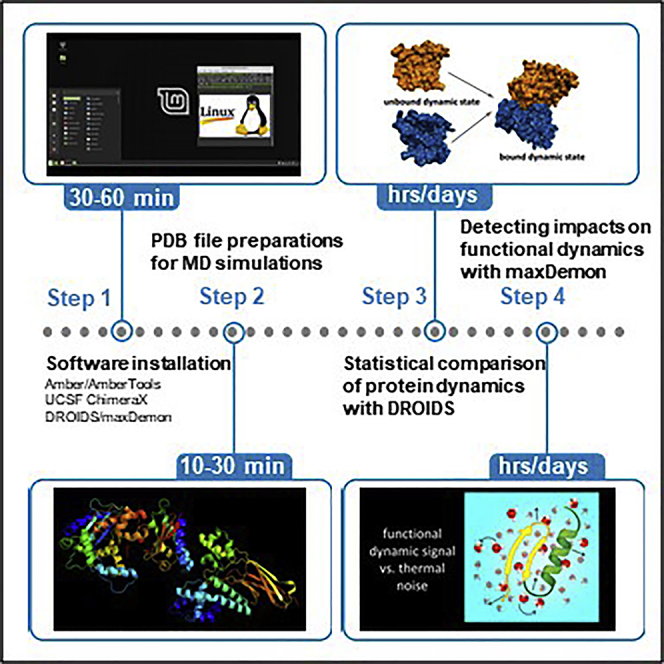

## Before you begin

### Software installation

**Timing: 30–60 min**

**Figure 1:**
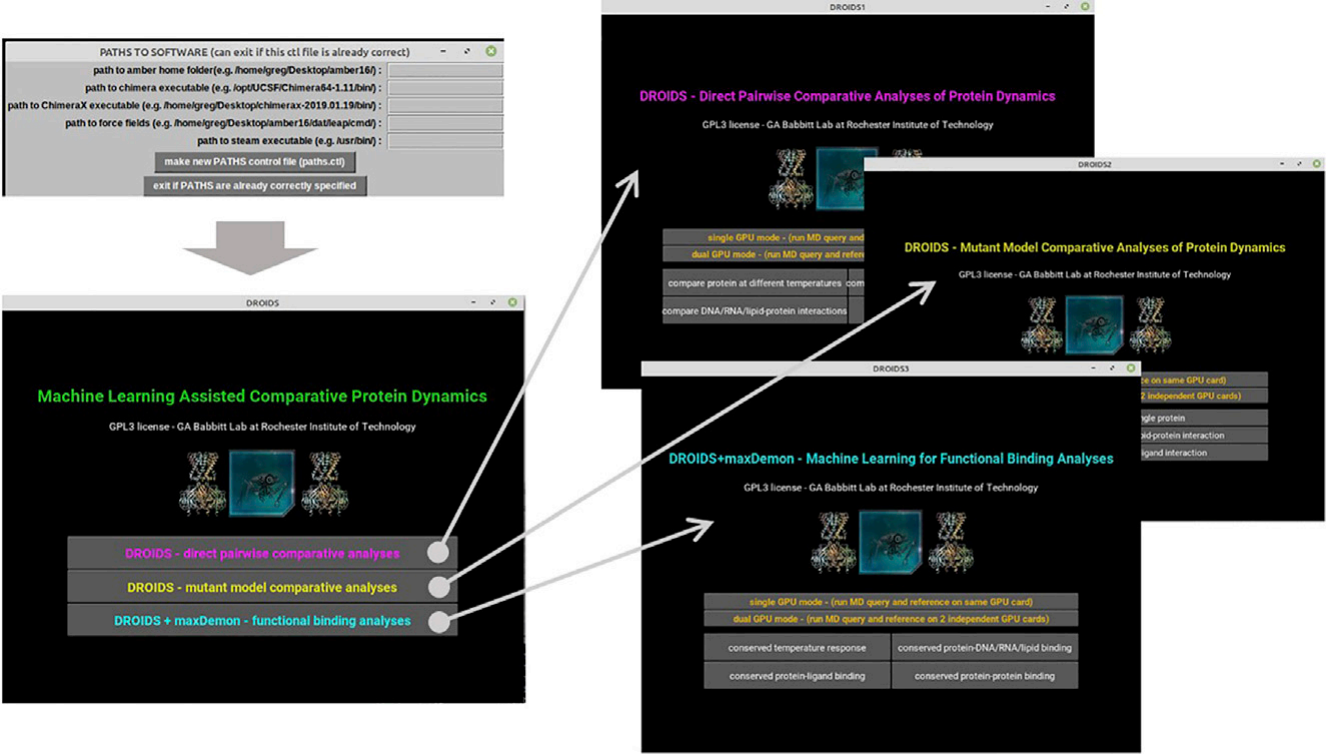
Graphical user interface (GUI) for analyses available using DROIDS pipeline for statistical comparison of dynamic motion The GUI are shown in the order in which they appear. The first GUI sets up paths to the relevant software dependencies (i.e., Amber and UCSF ChimeraX). It can be skipped if the control file was already previously made for your computer. The second GUI shows three main types of analyses to choose from. These are a simple statistical comparison of site-wise protein dynamics, a comparison of dynamics when mutations are introduced, and lastly, a comparison of site-wise dynamics followed by machine learning identification of conserved dynamics. Each button leads to one of three GUI where the basic type of functional comparison can be chosen. These include simple protein homologs, protein-DNA interaction, protein-small molecule ligand interaction, and lastly protein-protein interaction.

1. Before you begin you will need to have a desktop computer with a Linux OS (operating system) installed (as primary OS or as VM (Virtual Machine)) and 1 or 2 higher quality Nvidia graphics processing units (GPU) installed (e.g., GTX 1080, or RTX 2080/3080) along with appropriate Nvidia graphics drivers and CUDA, Nvidia’s parallel computing platform and application programming interface (API). Install main software dependencies and test the GPU as follows.
  a. Install UCSF Chimera and UCSF ChimeraX (Pettersen et al., 2004, 2021) for molecular visualization of protein structural files (i.e., PDB files).
    i. https://www.cgl.ucsf.edu/chimera/download.html
    ii https://www.rbvi.ucsf.edu/chimerax/download.html
  b. For molecular dynamics (MD) simulation, you will also need AmberTools (open source) as well as either a licensed installation of Amber (i.e., pmemd.cuda) (Case et al., 2005; Roe and Cheatham, 2013) OR an open source installation of python OpenMM.
    ***Note:*** We recommend the full licensed version of Amber/AmberTools as we have tested this more completely. Install and test MD runs on GPU as follows.
    i. https://ambermd.org/GetAmber.php
      Decompress and compile AmberTools into the Amber folder on the desktop first, then decompress and compile the licensed version of Amber into the same folder. All Amber code lives in this folder, so if/when it is deleted all Amber is removed from your system. After successfully compiling, write the following lines to the user’s bashrc file. Bashrc file can be opened at the terminal using the following command.
      $ cd
      $ xed .bashrc
      OR
      $ gedit .bashrc
      When the bashrc file opens, add the following lines to the bashrc file, then save and close file.
      ***Note:*** square brackets are to be substituted by user’s system information.
      source /home/[username]/Desktop/amber[version]/amber.sh
      export AMBERHOME=/home/[username]/Desktop/amber[version]/
      export PATH=$PATH:$AMBERHOME/bin
      test –f /home/[username]/Desktop/amber[version]/amber.sh
      Now the ‘make test’ command can be run from the terminal to test the Amber code. If you are building the GPU accelerated Linux system for this project, please be aware that the CUDA library folder on your system must be similarly sourced from bashrc. Please follow our installer code (below) or the CUDA installation instructions at Nvidia’s developer website for more guidance. https://developer.nvidia.com/
      Open amber folder at terminal and run test.
      $ cd $AMBERHOME
      $ make test
      ***Note:*** Most tests should be reported as PASSED if the GPU and Amber’s pmemd.cuda are working correctly. Floating point exceptions will be reported if you are not using a double point precision GPU (i.e., a gaming GPU like GTX or RTX instead of a Tesla GPU).
      ***Note:*** Our GitHub repo provides a perl script ‘AMBER_installer.pl’ to help users new to Linux navigate through the decompressing and compiling of Amber using Linux terminal commands.
      **CRITICAL:** If your computer has two GPUs that will be used to run the molecular dynamics simulations for the two-state comparison simultaneously, then find the two executables for pmemd.cuda_SPFP and pmemd.cuda_DPFP in the amber home directory on your desktop and create four copies named pmemd.cuda0_SPFP pmemd.cuda1_SPFP, pmemd.cuda0_DPFP, and pmemd.cuda1_DPFP.
  c. Install OpenMM (optional).
    i. https://openmm.org/

2. Download our custom pipeline and prepare folder to contain your MD comparative analysis. We also offer perl scripts for installing Amber/AmberTools and our DROIDS/maxDemon pipeline (AMBERinstaller.pl and DROIDSinstaller.pl) to simplify the following steps.
  a. Download DROIDS/maxDemon code folder https://gbabbitt.github.io/DROIDS-4.0-comparative-protein-dynamics/ and rename it as something simple (e.g., ‘DROIDS-4.4_pdbID’).
  b. Install Debian package and perl language package dependencies for DROIDS from terminal.
    $ sudo apt-get install [package]
    The Debian packages needed here are gedit, gdebi, evince, grace, perl-tk, python-tk, python-gi, python-kivy, libgstreamer1.0-0, and gstreamer1.0-plugins-base.
    Also install the perl package dependency as follows.
    $ sudo cpan App::cpanminus
    $ sudo apt install cpanminus
    $ sudo cpanm Statistics::Descriptive
  c. Install R language.
    $ sudo apt-get install r-base-core r-base r-base-dev
  d. Install R package dependencies at R console opened from Linux terminal.
    $ R
    > install.packages(‘[PackageName]’)
    Packages needed are ggplot2, gridExtra, dplyr, caret, FNN, e1071, kernlab, class, MASS, ada, randomForest, CCA, CCP, doParallel, foreach, rpsychi, and lattice.
  e. Then close the R console.
    > q()
  f. Go to the DROIDS folder, open a terminal, and run ‘python DROIDS.py’ in the terminal window. An introduction page will open, read and then close the Introduction page, and an initial GUI (Figure 1) will then prompt you to enter or exit the file paths for finding/running the software installed above. Suggestions for paths are printed to the terminal as well as a paths.ctl file. Please double check them. Copy this file onto your desktop for future DROIDS runs and you can later skip this step on subsequent runs by simply copying it with your PDB files into the DROIDS folders you set up in the future.
    ***Note:*** This file is specific to your machine and won’t work for jobs taken to other machines.
    A typical paths.ctl file might look similar to this.
    amber_path /home/[username]/Desktop/amber18/ # path to amber home folder
    chimera_path /opt/UCSF/Chimera64-1.11/bin/ # path to Chimera executable
    chimerax_path /home/[username]/Desktop/chimerax-2019.01.19/bin/ # path to ChimeraX
    teleap_path /home/[username]/Desktop/amber18/dat/leap/cmd/ # path to force field
    **CRITICAL:** Always keep the paths.ctl file in your working DROIDS folder.

### PDB file preparation

**Timing: 30 min to 1 h**

3. Before using our pipeline, you will need to find a PDB file for a protein structure that represents the natural functional comparison (e.g., protein unbound vs. bound to a drug/signaling molecule) or an evolutionary comparison (e.g., protein orthologs before and after a series of mutations, or protein paralogs representing a past gene duplication event) you wish to make. This will be cleaned up and converted into new files that represent its functional states of binding (e.g., 3 files representing the unbound kinase, the unbound ATP and well as the kinase-ATP complex). PDB files can be edited using a molecular visualization software such as UCSF Chimera,UCSF ChimeraX or PyMOL.
  ***Note:*** If a protein-ligand interaction is being studied, then create and save three PDB files (i.e., protein+ligand, protein only, and ligand only).
  a. Clean up the PDB files by removing all extraneous molecules (often used for crystallization) and any x-ray reflections (if present).
  b. Make sure no post-translational modifications are present that cannot be accounted for by the protein force fields in Amber. If such modifications are needed for study (e.g., glycosylation), remove them and then add them back using appropriate software such as https://glycam.org https://doi.org/10.1002/jcc.20820. When preparing the MD simulations, add an appropriate GLYCAM force field that is compatible with the protein force field that you are using (e.g., GLYCAM_06j-1 is only compatible with protein force fields ff12, ff12SB and later.).
  c. Crystallographic waters can be left in the file as they will be automatically removed during later steps of MD simulation preparation.
  d. Do not manually add hydrogens to the file as they will also be added automatically at a later step.
    ***Optional:*** Do add hydrogens manually if you intend to analyze a particular aspect of protonation. There will be an option to later skip reducing (i.e., add hydrogens) to the PDB file if you choose to set up the protonation manually.
  e. Open your PDB files with a text editor. Remove all line references to individual models (i.e., MODEL 1, MODEL 2, ENDMDL etc.).
    Examples of line references to be removed from PDB files.
    CONECT 1688 1711
    MODEL 2
    ATOM 1 O5 ' DC B 1 71.829 85.622 -69.018 1.00 62.33
    HETATM 2354 O HOH A1280 107.740 97.768 -63.768 1.00 55.97
    ENDMDL
    CONECT 316 312 315 317
    ***Note:*** If your files contain DNA structures, make sure that the amino acids of the protein structure are listed before the nucleotides, and then remove all lines with chain termination for the DNA (i.e., starting with TER) . Leave the TER lines for the protein chain(s). For example…
    ATOM 327 C4 DG B 16 71.673 123.072 -75.450 1.00 42.07
    TER 328 DG B 16 
    ATOM 329 O5' DC C 101 78.809 128.266 -68.196 1.00 63.12
    ***Note:*** If you are analyzing a protein-protein or protein-RNA/DNA interaction, you only need 2 PDB files for the reference protein in its bound and unbound states. If you are analyzing a protein-ligand interaction, you need the same two files (bound protein with ligand and unbound protein without the ligand) as well as an additional file with just the unbound ligand. This is because scaled quantum mechanical optimization must first be performed on the ligand, determining its bond characteristics so that the protein-ligand simulation can be executed.

## Key resources table

**Table:** 

REAGENT or RESOURCE	SOURCE	IDENTIFIER
**Software and algorithms**		

Linux OS on gaming PC Linux OS on gaming PC	Linux Ubuntu, or Mint websites	https://linuxmint.com/
Gaming compatible motherboard and PSU	Computer hardware dealers	https://www.newegg.com/
1-2 Nvidia GPU graphics processing units	Computer hardware dealers	https://www.nvidia.com/en-us/geforce/graphics-cards/30-series/rtx-3080-3080ti/
Nvidia CUDA install	Nvidia website	https://developer.nvidia.com/cuda-10.1-download-archive-base
Amber MD software	Amber MD project website	https://ambermd.org/
OpenMM software	OpenMM project website	https://openmm.org/
UCSF Chimera software	UCSF Chimera website	https://www.cgl.ucsf.edu/chimera/
UCSF ChimeraX software	UCSF ChimeraX website	https://www.rbvi.ucsf.edu/chimerax/
R statistical software project	Comprehensive R archive network	https://cran.r-project.org/
DROIDS/maxDemon software	Our website/repo	https://github.com/gbabbitt/DROIDS-4.0-comparative-protein-dynamics https://gbabbitt.github.io/DROIDS-4.0-comparative-protein-dynamics/

**CRITICAL:** If performing your own computer build, make sure your Linux Mint OS, CUDA versions, and Amber versions are compatible. Use versions released in roughly the same year.

***Note:*** If you intend to use open source OpenMM in place of a licensed version of Amber, you still need to download and install the open source AmberTools.

## Step-by-step method details

### Generating MD simulations for statistical comparison in DROIDS

**Timing: many hrs/days depending upon sampling and size of GPU**

This will lead you through the generation of molecular dynamics simulations for comparisons using the DROIDS GUI-based scientific pipeline.

**Figure 2:**
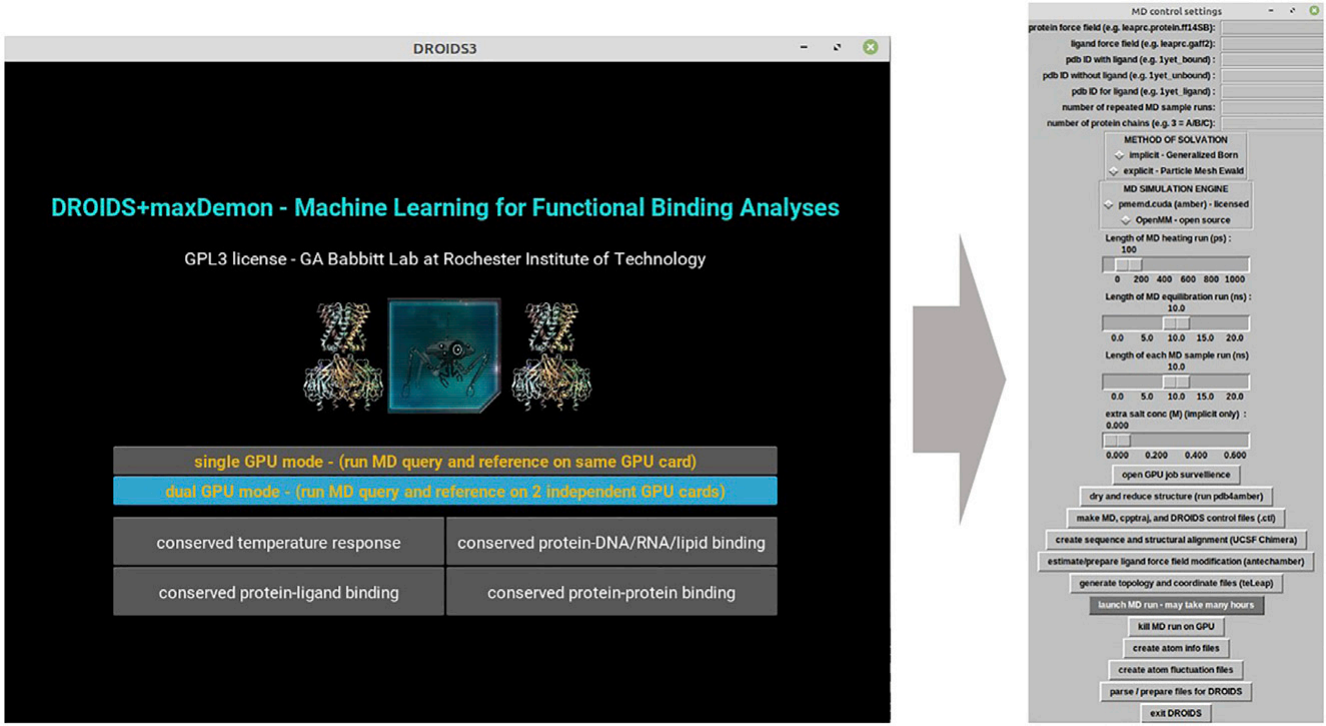
GUI for setup and control of Amber18/20 molecular dynamics simulations in DROIDS Here the user indicates whether the computer they are using has one or two graphical processor units (GPU), selects the type of functional comparison, and then opens the GUI for molecular dynamics control working from the top to the bottom of the GUI as indicated in the protocol.

1. From the main menu for DROIDS (Figure 1), please select one of several types of comparisons to run. If you intend to use maxDemon machine learning to detect functionally conserved regions of protein dynamics, then choose the third option.
2. Choose the type of model comparison you are running (e.g., simple protein, protein-DNA, protein-ligand or protein-protein interaction. See Figure 1). Be sure to also indicate whether your computer has one or two GPUs by highlighting the bar button on the GUI as shown below (Figure 2).

3. A GUI for running and post processing the molecular dynamics (MD) simulations will open and you will proceed pushing the buttons from the top to the bottom and following any additional instructions that appear in the Linux terminal. The control menu proceeds from top to bottom and the user proceeds linearly down the GUI (Figure 2).
  ***Note:*** We warn the potential user that appropriate quantitative and qualitative interpretations of the output should be based upon a good theoretical understanding of MD simulation and where it can go wrong. The biggest factors influencing the comparative outputs are the proper choice of modern force fields and the choice of an adequate sampling regime that will capture the scale of the physical phenomena one wants to observe. MD is notoriously slow as it is time stepped in femtoseconds. Most biological phenomena occur at nano or microsecond time scales. Therefore comparative protein dynamics should be applied to rapid phenomena that can be readily observed many times under nanosecond time scales. The comparison of atom fluctuation profiles of stable states are a good example of this, whereas comparison of longer term processes (e.g., involving protein folding of changes in conformational states) are not. A sampling regime of 100 production runs at 1 ns, with 10 ns of equilibration should be considered the bare minimum requirement for a statistically stable result. DROIDS should never be run only once, but several times to ensure that a comparison is stable and meaningful.
  The tasks to perform on the GUI from top to bottom are listed below
  a. (TEXT ENTRY) Enter PDB file names, force field file names, number of MD samplings (recommend = 100), and number of protein chains in the structure.
  b. (CHECKBOXES) Choose method of solvation (i.e., implicit OR explicit (preferred)) and MD simulation engine (i.e., Amber (preferred) OR openMM).
    ***Note:*** Comparison of MD with implicit solvation can run quite a lot faster but may not be as accurate as comparisons based upon explicit solvation.
  c. (SLIDERS) Set the sliders for length of MD runs (heating, equilibration, and production samples).
    ***Note:*** If the user wants to use range settings for the length of MD heating, equilibration, or production that are not available on the GUI, these can be manually edited in two files that control all the Amber MD settings (MDr.ctl and MDq.ctl for the reference and query protein structures resp.)
  d. (BUTTON: ‘open GPU job surveillance’) - opens top and nvidia-smi to watch CPU/GPU activity.
  e. (BUTTON: ‘dry and reduce structure’) - runs pdb4amber to add hydrogens (i.e., reduce), remove crystallographic waters (i.e., dry), reformat and resave PDB files.
  f. (BUTTON: ‘make control files’) – makes .ctl files that contain user inputs from above and are read by subsequent scripts in the analysis pipeline.
  g. (BUTTON: ‘create sequence and structural alignment’) - opens both PDB models in UCSF Chimera, and Linux terminal instructions guide user through superpositioning of models (via MatchMaker under Tools drop down menu) followed by structural alignment (via Match->Align). Make sure to save this alignment file in Clustal format (e.g., my_align.aln). When Chimera is closed, the user is prompted for the name of the saved .aln file and this is copied, renamed, and stored by the DROIDS program for later use.
  h. (BUTTON: ‘prepare ligand force field modification’) If protein-ligand model type was chosen earlier, this runs antechamber and sqm programs to generate mol2 file and force field modifications (.frcmod) for the ligand via scaled quantum mechanical optimization (logged in sqm.out).
  i. (BUTTON: ‘generate topology and coordinate files’) - runs teLeAP program to build the topology (.prmtop) and input coordinate (.inpcrd) files needed to set up both protein systems implicitly solvated in vacuum and charge neutralized in explicitly solvated octahedral water boxes. A .bat file is opened for each simulation and can be edited to modify the simulation preparations (e.g., change water box size, change water model, add additional force fields, load additional ligands etc.). When completed the DROIDS code will look for the output files and check their sizes to see that tLeAP has run to completion properly.
    **CRITICAL:** When done, the leap.log file and/or terminal outputs should be carefully read for any reported errors. Error at this stage will prevent MD simulations from launching.
  j. (BUTTON: ‘launch MD run’) launches the MD simulations and cycles through energy minimization, heating equilibration, and all MD production sampling runs. If the dual GPU option was chosen earlier, two new terminal windows will open and then close after simulations are completed. This process can take many hours or days to complete. Very large binary trajectory files (.nc) are created at this stage, often in the 10–100s GB size range. Please make sure there is enough space on the GPU. GPUs should be monitored on nvidia-smi to make sure that jobs queue properly (i.e., that only one pmemd0.cuda or pmemd1.cuda job runs on each card). If more than one job loads, close the terminals and use the pkill button right below to stop the GPUs. Then launch it again.
  k. (BUTTON: ‘create atom info files’) - creates atom information files using cpptraj.
  l. (BUTTON: ‘create atom fluctuation files’) - runs cpptraj on trajectory files to retrieve atom fluctuations and correlations.
  m. (BUTTON: ‘parse/prepare files for DROIDS’) - parses and analyzes these results for later analysis. Approximately 30 min. Also opens next GUI for statistical analyses to compare the sample sets of MD production runs.
  n. (BUTTON: ‘ exit DROIDS’) - optional button to exit DROIDS.

### Conducting statistical comparison in DROIDS

**Timing: 5–10 min**

**Figure 3:**
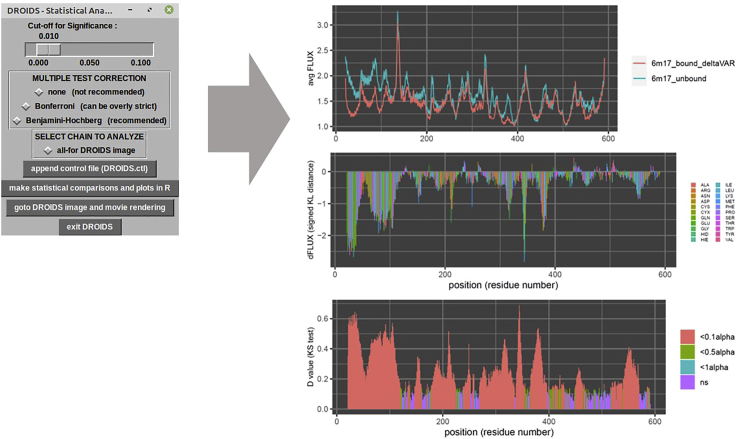
GUI and output for statistical comparison of dynamic motion This GUI produces R plot output for DROIDS analysis comparing the molecular dynamics of SARS-CoV-2 virus bound to human ACE2 receptor domain to that of unbound human ACE2 receptor domain (PDB 6M17).

This next GUI will produce output data files and R plots of results in a DROIDS output folder (Figure 3).

4. Select alpha level for the tests and a method of correction for multiple tests.
  ***Note:*** This correction is necessary to control for the fact that a statistical hypothesis test is conducted for every amino acid site in the comparison. Benjamini-Hochberg is recommended. If models have more than one protein chain, options to analyze and plot each single chain will also appear.
  a. Button creates a control file to store this information (DROIDS.ctl).
  b. Button runs site-wise statistical tests, and R plot (Figure 3) will appear to show positional atom fluctuations along protein sequence for both models, differences in atom fluctuation along the sequence (as dFLUX = signed symmetric Kullback-Leibler or KL divergence), and statistical significance of these dynamic differences along the sequence (as determined via 2 sample Kolmogorov-Smirnov or KS test). See Equation 3 in (Babbitt et al., 2020a), Equation 4 in (Babbitt et al., 2020b), and Figure 2 from (Rynkiewicz et al., 2021)
  c. Button to open GUI for molecular visualization of these results.
  d. Optional button to exit DROIDS.

### Color mapping images and movies of comparative dynamics in DROIDS

**Timing: 10–30 min**

**Figure 4:**
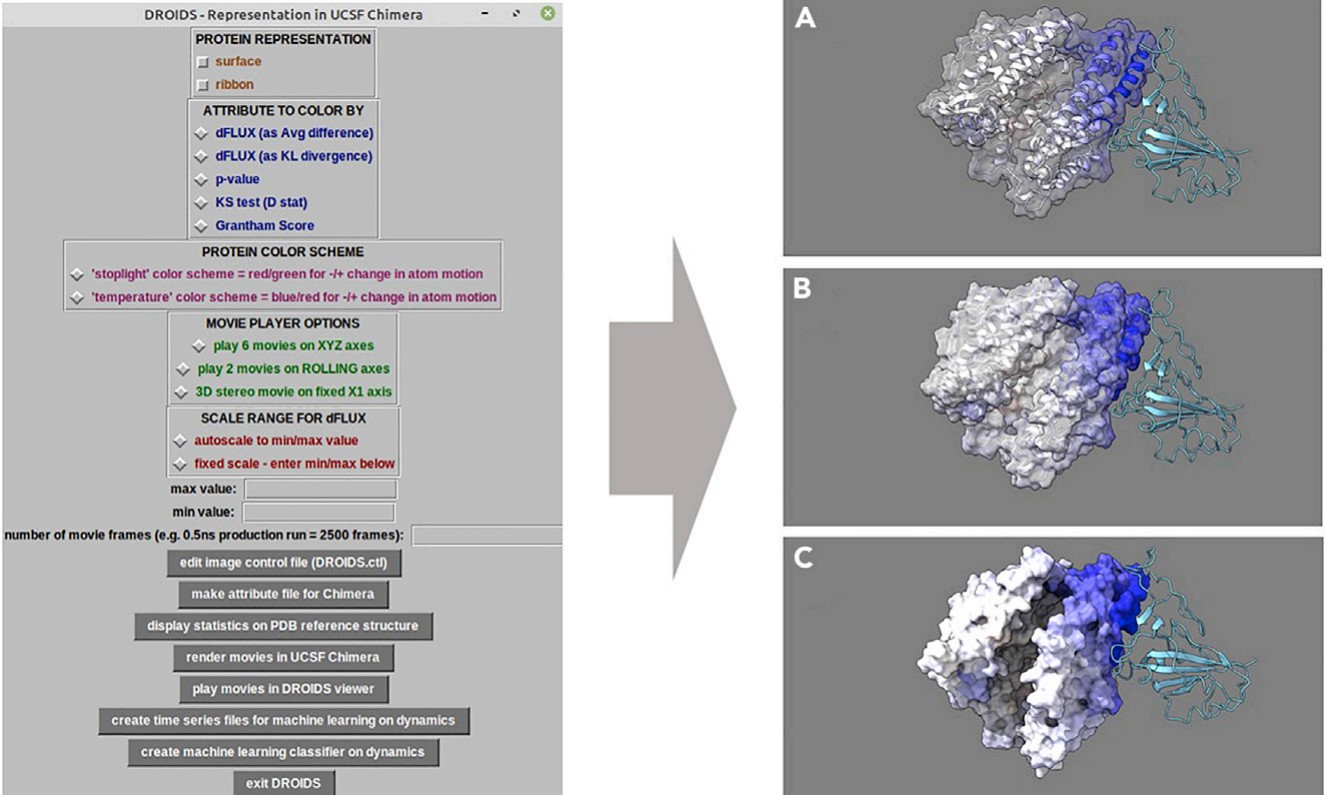
GUI and output for visualization of divergence in dynamics This GUI produces color mapping for DROIDS analysis showing dampened atom fluctuation as negative KL divergence (blue) caused by binding of SARS-CoV-2 (aqua ribbon) to human ACE2 receptor domain (PDB 6M17). Three levels of decreasing transparency are shown (A = 80%, B = 50% and C = 0%). The last image also shows UCSF ChimeraX full ambient occlusion lighting. Supplemental movie files (Videos S1 and S2 show spin movie of KL divergence and associated p-values for the site-wise KS test. Red = significant dampening of motion, black = non-significant change in dynamics).

This GUI allows the positional information regarding the statistical comparison of the protein dynamics to be color-mapped to a reference PDB file and to short movies of the dynamics (Figure 4). We recommend you start with KL divergence since averaging can reduce the information in the color mapping. See our software note for details regarding the different metric. Please refer to (Babbitt et al., 2018, 2020a, 2020b; Rynkiewicz et al., 2021)

5. (CHECKBOXES) Select protein visual model, color scheme, statistical attributes, and color scaling mode for visualization of DROIDS results in UCSF ChimeraX window.
  a. (BUTTON: ‘edit image control file’) - edits DROIDS.ctl with the options selected here.
  b. (BUTTON: ‘make attribute files for Chimera’) - creates ‘color by attribute’ files in the proper format needed by UCSF ChimeraX.
  c. (BUTTON: ‘display statistics on PDB reference structure’) - opens selected color mappings in UCSF ChimeraX display (Figure 4).
    ***Note:*** Images and spin movies of color-mappings can be generated and saved from the UCSF ChimeraX GUI. This GUI can be closed and reopened repeatedly to generate visuals of all the various options (avg. difference, p values, KL divergence etc.). Video S1 shows a spin representation of the SARS-CoV-2 viral binding signature on ACE2 in terms of KL divergence. Degree of blue indicates degree of atom fluctuation dampening under binding. Video S2 shows significant p-values for changes in dynamics under viral binding to ACE2 in red. Black is not significant.
  d. (BUTTON: ‘render movies in UCSF Chimera file’) - allows color-mapped movies of the dynamics to be rendered in UCSF Chimera.
    ***Note:*** If surface models are used, the rendering process is significantly slowed.
  e. (BUTTON: ‘play movies in DROIDS viewer’) - After rendering, movies are played back from ‘Movie Player Options’ using customized gstreamer movie players.
  f. At this point the DROIDS analyses are complete. If the maxDemon machine learning option was chosen on the main GUI at the beginning of the pipeline, two more buttons remain. The first will parse the molecular dynamics data into many time slices for machine learning, while the second will open the maxDemon GUI (see next section).
  g. (BUTTON: ‘exit DROIDS’) - Optional button to exit DROIDS.

Video S1. Spin video showing color mapping for KL divergence from DROIDS analysis (step 11)

Video S2. Spin video showing color mapping for p values from DROIDS analysis (step 12)

### Detecting functionally conserved protein binding dynamics and comparing genetic/drug class variant impacts on conserved dynamics in maxDemon

**Timing: 1–2 days**

At this stage, users can further utilize the multi-agent machine learner (maxDemon) to parse and display the regions with evolutionary conserved dynamics (i.e., nanomachine-like motions) involved in the functional binding interactions from the random thermal noise that is also present in the MD simulations. The analyses can also compare genetic or drug class variant impacts on the conserved dynamics (optional), and map sites with coordinated motions within the conserved dynamics.

**Figure 5:**
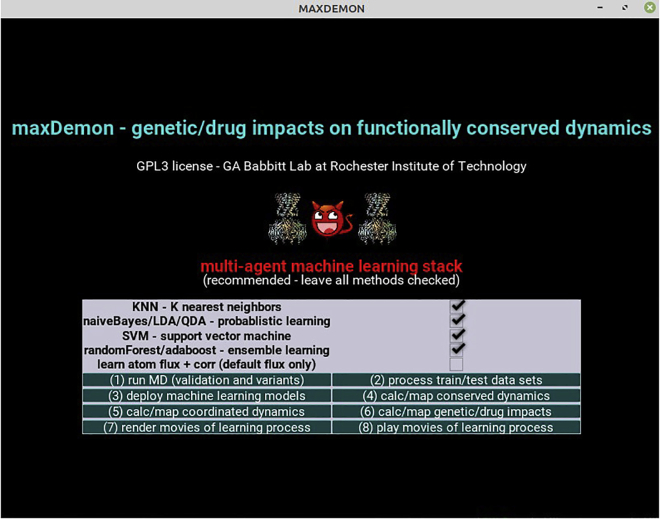
GUI for maxDemon machine learning identification and visualization of conserved dynamics Here, the user selects the machine learning methods to be stacked into the multi-agent learner. Then the user follows the numbering of the buttons on the GUI as outlined in the protocol.

**Figure 6:**
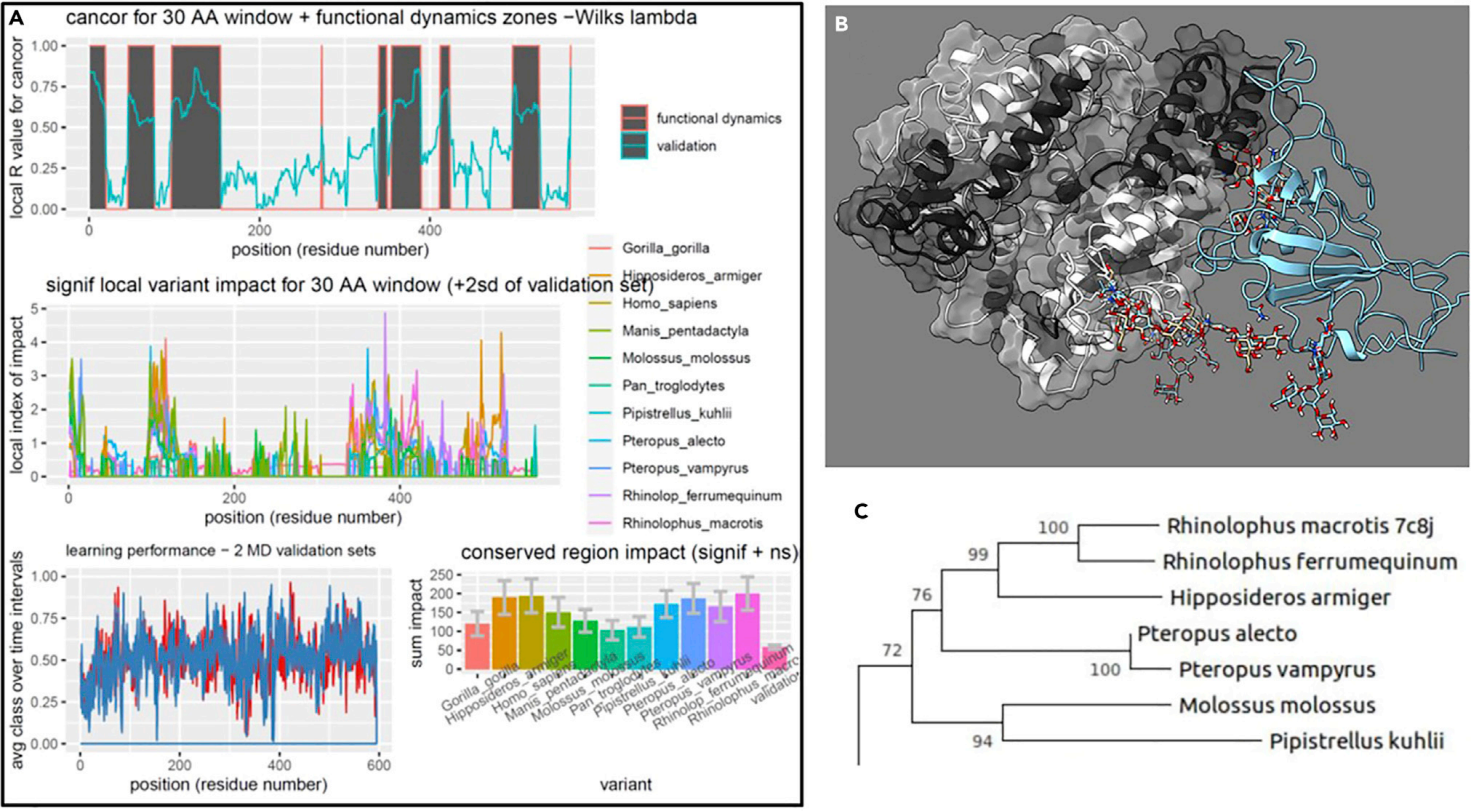
Output for machine learning identification and visualization of conserved dynamics (A) The maxDemon analysis output is shown in (A) and comprises identification of functionally/evolutionary conserved dynamic regions (dark gray), the local and total conserved region impacts of genetic variants representing human, ape and various bat species orthologs of ACE2 protein. Error bars on the conserved region impacts represent 95% confidence intervals for 100 bootstrap replications. (B) Color mapping for maxDemon analysis showing conserved dynamics (dark grey) caused by binding of SARS-CoV-2 (aqua ribbon) to Rhinolophus bat ACE2 receptor domain (PDB 7c8j). (C) A maximum likelihood tree representing the bat orthologs generated in MEGAX (Kumar et al., 2016). Video S3 shows spin representation of image in (B).

**Figure 7:**
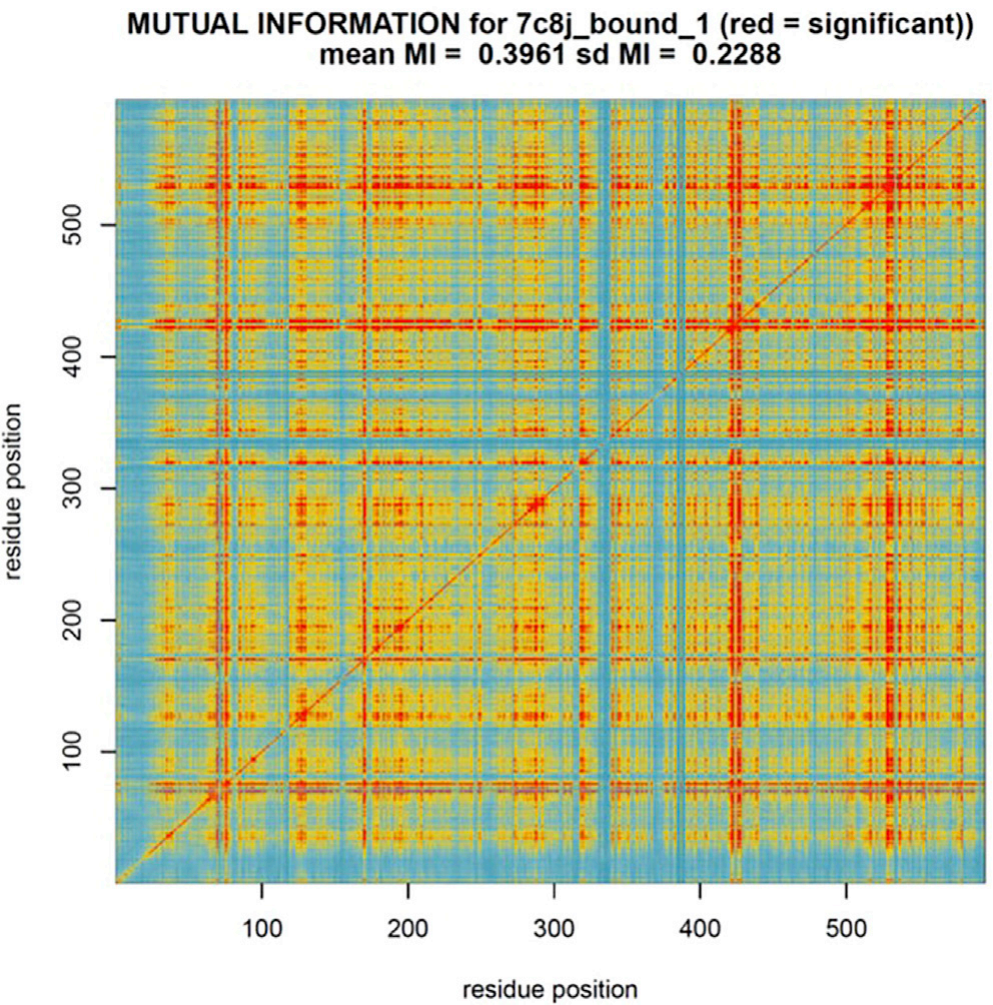
Output for visualization of coordinated site dynamics A heatmap showing levels of coordinated dynamics determined by mutual information in learning classifications over time in the validation MD run. Blue denotes no coordination, yellow denotes moderate coordination and red denotes high coordination.

6. Check or uncheck the settings for this analysis (Figure 5).
  a. Select up to 7 machine learning methods to be used to classify bound vs. unbound protein dynamics.
    ***Note:*** The machine learner(s) are trained upon the simulations generated earlier using DROIDS and then deployed upon new simulations generated at this stage.
    ***Note:*** Support vector machines can be very slow on large sampled MD systems. An option to subsample the data to speed the analysis is given at the Linux terminal if the SVM option is chosen. A kernel method (e.g., linear, polynomial or radial basis function) must also be selected at the terminal if the SVM option is chosen.
  b. A multivariate feature vector for machine learning is now created comprised of small time slices of atom fluctuations at a given amino acid site/position as well as fluctuations at nearby sites 1,3, and 9 positions downstream on the protein backbone. One can also select to analyze correlations of the motion at these downstream sites to the given amino acid site/position. See Equation 4 from (Babbitt et al., 2020a) and Figure 3 from (Rynkiewicz et al., 2021).

7. Push the buttons on the maxDemon GUI in the order in which they are numbered (Figure 5).
  a. (1) This button opens a GUI to set up and run MD simulations upon which the machine learning will be deployed.
    ***Note:*** It is run similarly to the GUI for MD runs used earlier in DROIDS. At minimum 3 new simulations are run on the functionally bound state of the protein. Two of these are just copies of the functionally bound reference PDB file. The third must be an evolutionary ortholog of the protein in its functionally bound state. This ortholog can be found at Protein Data Bank OR it can be modeled from the reference PDB in UCSF Chimera using the swapaa function. Initially, a series of text files are opened in which the user will list the names of the PDB files to be put to MD simulation and machine learning analysis (i.e., the two validation runs, the ortholog run, then all runs for the genetic/drug class variants to be compared). If protein-ligand option is used, two text files listing the unbound protein structures and their respective ligands will be prompted. The length of the MD runs can be set to 1×, 3×, 5× or 10× the length of a single production run in the DROIDS training set. Buttons on this GUI make control files (.ctl) for this part of the analysis, dry and reduce PDB files, create additional alignments if needed (optional), create force field modifications for additional ligands (if needed), create topology (.prmtop) and input coordinate files (.inpcrd) files for the MD simulation, launch the MD simulations, create atom information files, calculate atom fluctuation and atom correlations in cppraj, and parse the results for later steps.
  b. (2) Button parses and processes training/testing data sets for R implementation.
  c. (3) Button runs machine learning in R over each consecutive method chosen and each site on the protein. A multi-agent classifier is constructed using K nearest neighbors, naïve Bayes, linear and quadratic discriminant function analyses, support vector machine, random forest, and adaptive gradient boosting (AdaBoost).
  d. (4) Button uses canonical correlation analysis between the machine learning profiles in the two validation MD runs and the ortholog MD run to detect protein regions with significantly similar functional/evolutionary conserved dynamics (i.e., not due to thermal noise). See Equation 6 from (Babbitt et al., 2020a), Equations 3 and 4 from (Babbitt et al., 2020b) and Figure 3 from (Rynkiewicz et al., 2021). These conserved regions are color mapped in the R plots and PDB structure in UCSF ChimeraX using dark gray (Figure 6, Video S3). This analysis typically indicates regions of the protein with important functional motion that may be conserved over deep evolutionary time scales. A relative entropy calculation is used to quantify the impacts of the genetic or drug class variants on the conserved dynamics identified by the learner(s) in the comparative MD simulations. See Equation 7 from (Babbitt et al., 2020a), Equation 5 from (Babbitt et al., 2020b), and Equation 3 and Figure 4A from (Rynkiewicz et al., 2021)
  e. (5) Sites of coordinated machine learning classifications over time are determined via a calculation of mutual information and displayed in a heat map (if Wes Anderson color palette is chosen at the Linux terminal, then blue = no coordination, yellow= moderate coordination, and red= high coordination). See Figure 7. Also see Equation 4 and Figure 4 (Rynkiewicz et al., 2021) for more mathematical details.

Video S3. Spin video showing color mapping for conserved dynamics regions from DROIDS/maxDemon analysis (steps 13 and 14)

## Expected outcomes

The expected outcomes are the creation of DROIDS results and a maxDemon results folder that contains the PDF file of the multi-panel plots in Figures 3 and 6, and the heatmap in Figure 7, as well as the color-mapped structures in Figures 4 and 6. These color-mappings are automatically opened in UCSF ChimeraX whereupon the user can rotate and zoom the molecular view and save images. The user can also create spin movie images from the ChimeraX toolbar as well.

## Limitations

Limitations of the software are governed by the proper application of molecular dynamics simulations to any given problem, the limitations of the force field parameters chosen for a given system, and proper sample size and sampling times. For most comparative protein analyses, we recommend using explicit solvation and generating a sample of at least 100 MD production runs for at least 1 ns per run. We recommend at least 10 ns of equilibration as well. DROIDS and maxDemon analyses should always be run more than once to assure that the quantitative and qualitative results are stable. We warn the potential user that appropriate quantitative and qualitative interpretations of the output should be based upon a good theoretical understanding of MD simulation and where it can go wrong. The biggest factors influencing the comparative outputs are the proper choice of modern force fields and the choice of an adequate sampling regime that will capture the scale of the physical phenomena one wants to observe. MD is notoriously slow as it is time stepped in femtoseconds. Most biological phenomena occur at nanosecond or microsecond time scales. Therefore comparative protein dynamics should be applied to rapid phenomena that can be readily observed many times under nanosecond time scales. The comparison of atom fluctuation profiles of stable states are a good example of this, whereas comparison of longer term processes (e.g., involving protein folding of changes in conformational states) are not. DROIDS should never be run only once, but several times to ensure that a comparison is stable and meaningful.

## Troubleshooting

### Problem 1

Alignment files are gibberish.

### Potential solution

Do not use nested names for PDB files (e.g., myfile.pdb and myfile_alt.pdb). Use distinctly different names instead (e.g., myfileA.pdb and myfileB.pdb). Our scripts often use pgrep or RegEx match statements to find file names in text.

### Problem 2

During atom fluctuation calculations a file is reported missing.

### Potential solution

Rerun MD simulations again in step 3 j. An MD production run job was likely interrupted at some point, possible due to lack of space on the GPU.

### Problem 3

MD runs are all being aborted as they queue and reporting memory errors.

### Potential solution

Amber has not been properly installed and tested on the GPU or a GPU has failed and needs to be replaced. Run tests on the GPU as described on the Amber websites. Your molecular system may also simply be too large (i.e., too many parameters) for your GPU hardware.

### Problem 4

The main GUIs will not open due to python-kivy dependency problems on your machine.

### Potential solution

Use the alternative and more stable GUIs by typing ‘perl DROIDS.pl’ and ‘perl maxDemon.pl’ instead of ‘python DROIDS.py’ and ‘python maxDemon.py’ at the Linux terminal. Otherwise check all python dependencies and reinstall python-kivy watching for error messages during the install process.

## Resource availability

### Lead contact

Further information and clarification should be directed to and will be fulfilled by the lead contact, Dr. Gregory A. Babbitt (gabsbi@rit.edu).

### Materials availability

There are no materials needed for this protocol.

## Data Availability

Our pipeline code is available at the following website and GitHub repository: https://gbabbitt.github.io/DROIDS-4.0-comparative-protein-dynamics/ https://github.com/gbabbitt/DROIDS-4.0-comparative-protein-dynamics
